# Sex differences in apoptosis do not contribute to sex differences in blood pressure or renal T cells in spontaneously hypertensive rats

**DOI:** 10.3389/fphys.2022.1006951

**Published:** 2022-10-11

**Authors:** Mahmoud Abdelbary, Riyaz Mohamed, Ellen E. Gillis, Karl Diaz-Sanders, Babak Baban, Michael W. Brands, Jennifer C. Sullivan

**Affiliations:** ^1^ Department of Physiology, Augusta University, Augusta, GA, United States; ^2^ Department of Oral Biology, Augusta University, Augusta, GA, United States

**Keywords:** kidney, cell death, necrosis, inflammation, gender

## Abstract

Apoptosis is a physiological and anti-inflammatory form of cell death that is indispensable for normal physiology and homeostasis. Several studies have reported aberrant activation of apoptosis in various tissues at the onset of hypertension. However, the functional significance of apoptosis during essential hypertension remains largely undefined. The current study was designed to test the hypothesis that apoptosis contributes to sex differences in blood pressure and the T cell profile in spontaneously hypertensive rats (SHR). Apoptosis was measured in kidney, aorta and spleen of 13-week-old adult hypertensive male and female SHR. Female SHR had greater renal and aortic apoptosis compared to age-matched males; apoptosis in the spleen was comparable between the sexes. Based on well-established sex differences in hypertension, we tested the hypothesis that greater apoptosis in female SHR contributes to the lower BP and pro-inflammatory profile compared to males. Male and female SHR were randomized to receive vehicle or ZVAD-FMK, a cell permeable pan-caspase inhibitor, in established hypertension from 13 to 15 weeks of age or at the onset of hypertension from 6 to 12 weeks or age. Treatment with ZVAD-FMK lowered renal apoptosis in both studies, yet neither BP nor renal T cells were altered in either male or female SHR. These results suggest that apoptosis does not contribute to the control or maintenance of BP in male or female SHR or sex differences in renal T cells.

## 1 Introduction

Hypertension is a primary risk factor leading to multiple cardiovascular diseases, including stroke and myocardial infarction. Based on the most recent guidelines defining hypertension, approximately half of adults in the United States are hypertensive (Whelton et al., 2018). Despite receiving up to 4 different classes of anti-hypertensive medications, blood pressure (BP) remains poorly controlled in many patients (Hajjar and Kotchen, 2003). Therefore, better understanding of the mechanisms contributing to pathogenesis of hypertension will help identify new therapeutic targets to reach the desired BP goals and improve the quality of life of patients with poorly controlled BP.

Over the past decade, an increasing body of literature has highlighted the contribution of various components of the immune system to the control of BP and the pathogenesis of hypertension in both animal models of hypertension and in humans (Drummond et al., 2019). In particular, T cells have been implicated in the development and maintenance of hypertension (Madhur et al., 2021). However, key questions remain in the field regarding how the immune system is regulated and T cells are activated in hypertension. Of interest, cell death is a primary factor contributing to modulation of the immune system (Nagata and Tanaka, 2017; Yatim et al., 2017). In particular, necrotic cell death can promote inflammation via the release damage-associated molecular patterns which induce the activation of dendritic cells, macrophages and other sentinel cells of the innate immune system, with sustained necrosis leading to T cell activation (Martin, 2016). Indeed, we recently published that necrotic cell death contributes to the pro-inflammatory T cell profile in male and female spontaneously hypertensive rats (SHR). However, necrotic cell death contributes to the development of hypertension only in males (Abdelbary et al., 1979). Cell death can be categorized into both an immunogenic or inflammatory form of cell death, necrosis, or a non-immunogenic and anti-inflammatory form of cell death, apoptosis. Apoptosis, also known as caspase-mediated programmed cell death, is a well-known physiologic form of cell death that is essential for embryonic development and maintenance of homeostasis (Szondy et al., 2017; Galluzzi et al., 2018). Of interest, several studies have reported a significant increase in apoptotic cell death in multiple organs during hypertension, including the kidney, brain, heart and blood vessels (Fortuño et al., 1979; Hamet et al., 1979; Jiang et al., 2013). However, the contribution of apoptosis to the pathogenesis of hypertension or the control of BP is unknown. Since apoptosis does not induce an inflammatory response, and in some cases is anti-inflammatory (Szondy et al., 2017), it is conceivable that increases in apoptosis could limit T cell activation and subsequent increases in BP.

There are well-established sex differences in hypertension in SHR where females have a lower BP compared to age-matched male SHR (Elmarakby and Sullivan, 2021). Several studies have also reported greater apoptotic cell death in females compared to males in various organs and cell types under both physiological (Tsukahara, 2009; McCarthy et al., 2015) and pathological conditions (Li et al., 2005; Lang and McCullough, 2008). Neurons and bone marrow derived macrophages isolated from females tend to die predominantly via apoptosis when subjected to stress, while cells from males preferentially die via necrosis (Du et al., 2004; Jog and Caricchio, 2013). In addition, greater apoptosis in females is associated with less damage and a better prognosis in animal models of ischemic stroke (Li et al., 2005; Lang and McCullough, 2008).

The first goal of the current study was to determine if female SHR have greater apoptosis compared to males. We report greater apoptotic cell death in both thoracic aorta and kidney of female SHR compared to males. Based on the potential anti-inflammatory effects of apoptosis (Szondy et al., 2017), we next hypothesized that greater apoptotic cell death in females contributes to their lower BP and more anti-inflammatory renal T cell profile. However, in contrast to our hypothesis, inhibition of apoptosis did not change BP or the T cell profile in male or female SHR.

## 2 Materials and methods

### 2.1 Animals

Male and female SHR (Envigo, Inc. Indianapolis, Indiana) were used in all studies. All experiments were conducted in accordance with the National Institutes of Health Guide for the Care and Use of Laboratory Animals and approved and monitored by the Augusta University Institutional Animal Care and Use Committee. Animals were housed under conditions of constant temperature and humidity and exposed to a 12:12-h light-dark cycle. All rats were given free access to rat chow and tap water. Initial studies were performed in 6 and 13 week old male and female SHR (*n* = 6). At the end of all studies, rats were anesthetized with isoflurane and a thoracotomy was performed. Terminal blood samples were obtained via aortic puncture. Kidney, thoracic aorta and spleen were harvested for flow cytometric analysis of cell death and T cells.

Additional 11 week old male and female SHR were implanted with telemeters for the measurement of mean arterial blood pressure (MAP) as previously described (*n* = 4–8) (Pollock and Pollock, 2001). Rats were allowed 1 week to recover from surgery. Baseline BP was then measured for 5 days. To determine the effect of apoptosis on BP in established hypertension, adult, hypertensive male and female SHR were randomized to treatment with vehicle (2% dimethyl sulfoxide in deionized water) or ZVAD-FMK (Wang et al., 2015) (1 mg/kg; Enzo Life Sciences) *via* intraperitoneal (IP) injection 5 days a week from 13 to 15 weeks of age. ZVAD-FMK is a non-cytotoxic cell permeable inhibitor of apoptosis that works by irreversibly binding the catalytic sites of the caspase serine proteases which are known to function as both initiators and executers of apoptosis (Slee et al., 1996).

To determine the contribution of apoptosis to the development of hypertension, 6 week old male and female SHR were randomized to vehicle (2% dimethyl sulfoxide in deionized water) or ZVAD-FMK (1 mg/kg) treatment via intraperitoneal (IP) injection 3 days a week (on Saturday, Monday and Wednesday) until 12 weeks of age (*n* = 7). Based on laboratory experience, female SHR do not survive telemetry implantation prior to 9 weeks of age. Therefore, for this study, BP was monitored weekly via tail-cuff plethysmography as previously described (Pollock and Pollock, 2001). At the end of the study, a section of aorta was harvested for histological assessment of fibrosis and wall thickness.

### 2.2 Flow cytometric analysis

**FIGURE 1:**
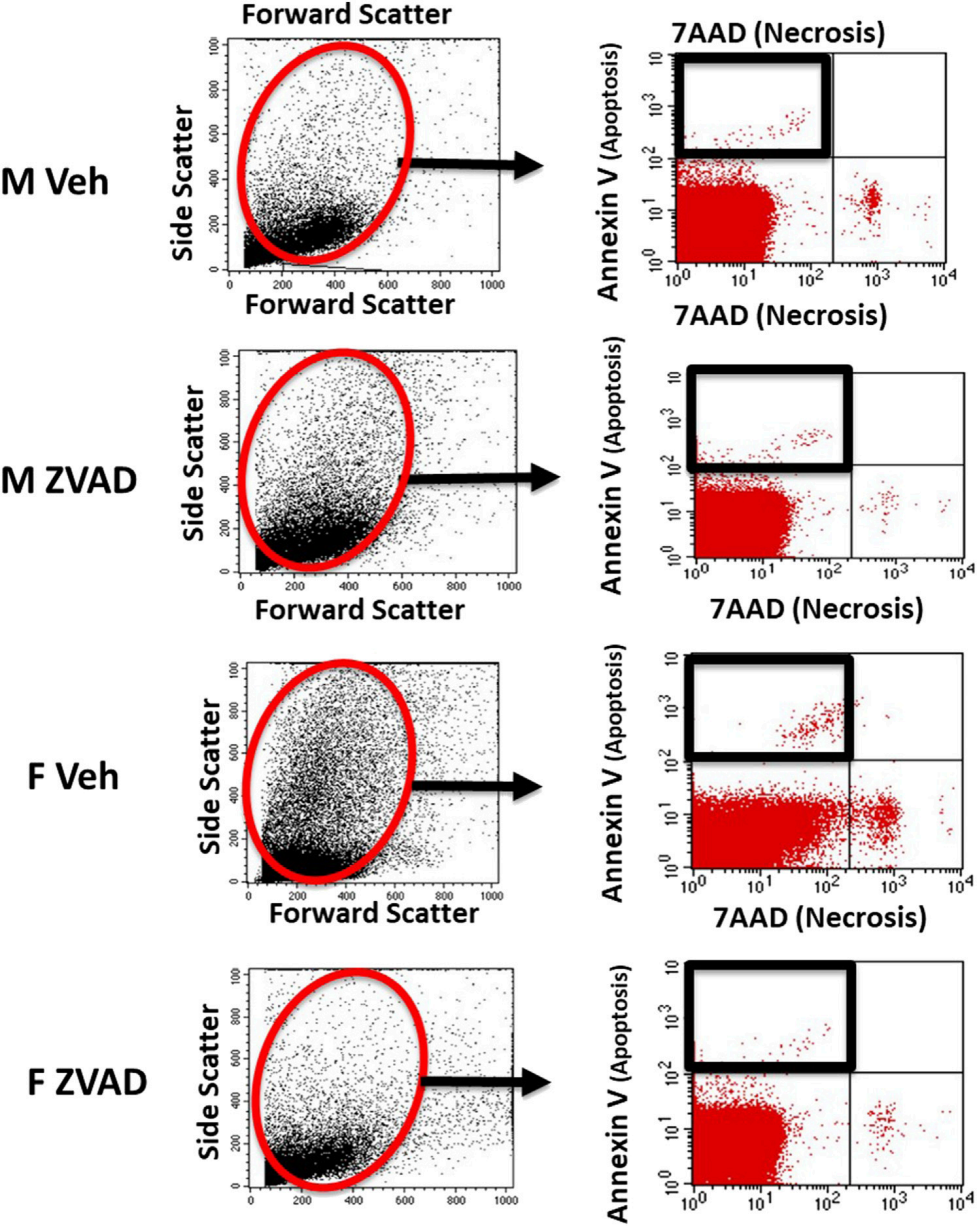
Apoptosis was analyzed by flow cytometry. Shown are representative scatterplots of flow cytometry gating strategy showing the selection of single cells and exclusion of cell debris based on forward scatter and side scatter.

Single cell suspensions of kidneys, spleen and thoracic aorta with intact perivascular adipose tissue were prepared in RPMI 1640 media (Corning Life Sciences; Catalogue: 17-195-CV) containing 10% fetal bovine serum as previously described (Tipton et al., 2014). Apoptotic cell death was measured by flow cytometry in single cell suspensions in untreated, control male and female SHR using a PE Annexin V Apoptosis Detection Kit I according to the manufacturer instructions (BD Biosciences, San Diego, CA). Apoptosis and necrosis were measured in vehicle and ZVAD-FMK-treated male and female SHR using a PE Annexin V Apoptosis Detection Kit I and 7AAD according to manufacturer instructions (BD Biosciences, San Diego, CA). Representative staining and flow cytometry gating for apoptosis is shown in Figure 1.

**FIGURE 2:**
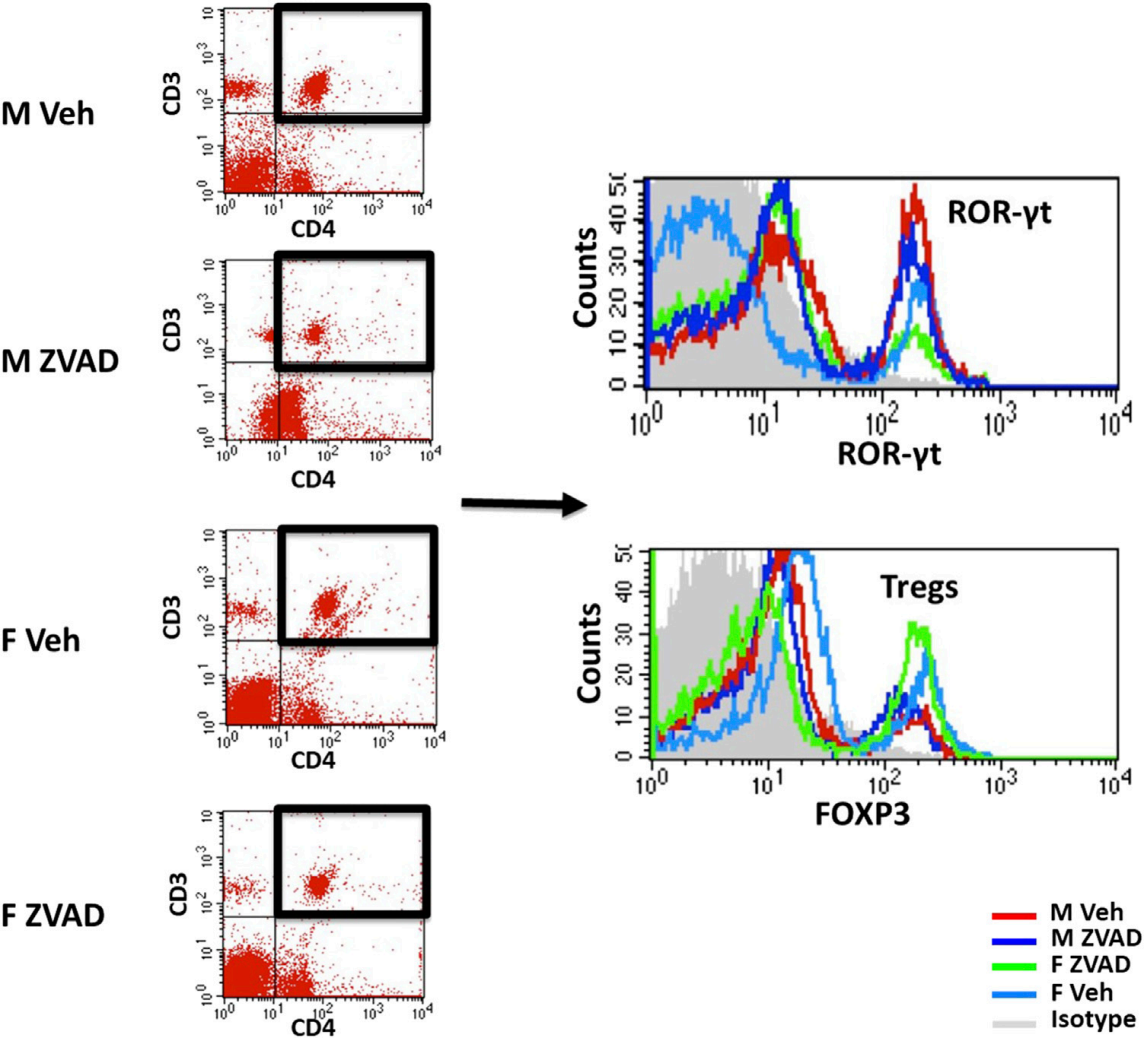
T cell profiles were analyzed by flow cytometry. Shown are representative scatterplots of flow cytometry gating strategy showing the selection of single cells and exclusion of cell debris based on forward scatter and side scatter. T cells were analyzed based on the expression of CD3 and/or CD4. Total CD3^+^CD4^+^ T cells were further gated for expression of ROR-γt for T helper 17 cells or Foxp3 for Tregs.

Phenotypic analyses of T cells in kidney and spleen were performed as previously described (Tipton et al., 2012). Cells were incubated with cell surface antibodies for CD3 (1:100; eBioscience; 46-0030-82) and CD4 (1:100; eBioscience; 554837) for 20 min on ice in the dark. Cells were then washed, fixed and permeabilized (eBioscience; 00-5523-00) and then stained with intracellular antibodies for FoxP3 (1:100; eBiosciences; 17-5773-82), or RAR-related orphan receptor-γ (RORγ; 1:100; R&D Systems; IC6006P) to identify Tregs (CD3^+^CD4^+^FoxP3^+^) and T helper (Th)17 cells (CD3^+^CD4^+^RORγ^+^), respectively. Samples were run on a four-color flow cytometer (FACS Calibur, BD Biosciences) and data were analyzed using Cell Quest software. Single color controls were used to set compensation. Antibody specificity was confirmed using isotype controls. Representative staining and flow cytometry gating for T cells is shown in Figure 2.

### 2.3 Western blot

Using a razor blade, transverse sections of 50 mg were made in frozen kidneys to include all 3 regions (cortex, outer medulla and inner medulla). Frozen sections were immediately suspended in 1 ml RIPA lysis buffer (Pierce RIPA buffer; Thermo Scientific; 89901) containing freshly added protease and phosphatase inhibitor cocktails (Halt protease inhibitor cocktail, 78429; Halt phosphatase inhibitor, 78428). Sections were homogenized for 30 s on ice using a tissue homogenizer (ThemoFisher; 15340167) followed by sonication at 50% pulse, 10 times for 2 s each on ice. Tissue lysates were then centrifuged at 14,000 g for 10 min and the supernatant were collected for protein estimation using a Bradford protein assay (Quick start Bradford 1x dye reagent; Bio-Rad; 5000205) as per manufacturer instructions. Samples for Western blotting were prepared using 2X lamelli buffer (BioRad; 1610737) containing freshly added β-mercapto-ethanol. Samples were boiled at 95°C for 10 min then loaded onto a precast gradient gel (BioRad; 4561096; 25 µg/well). Gels were run on a Bio-Rad Powerpac 300 at ∼120 V and proteins were transferred onto polyvinylidene difluoride membranes (Immobilion FL PVDF; Millipore sigma; IPFL00010) using a Bio-Rad Powerpac 200 at 100 V for 1 h on ice. Membranes were then washed and blocked in 5% bovine serum albumin (BSA; Sigma; A9418) in tris buffered saline containing 0.1% tween 20 (TBST) for 1 h. Membranes were blotted against caspase-3 (Cell signaling catalog number 9662; 1:500 dilution in 5% BSA in TBST) incubated for 48 h at 4°C. Equal protein loading was confirmed by probing for β-actin for 1 h at room temperature (Sigma Aldrich catalog number A1978; 1:10000 dilution in 5% BSA in TBST). Cell Signaling confirmed the specificity of the antibody for caspase-3 using CASP3 knock-out HCT116 cells. Protein expression was visualized using the appropriate IRDye secondary antibody at 1:10,000 dilution in 5%BSA in TBST for 1 h at room temperature (LI-COR; 92632210 and 92668072). Specific protein bands were detected using the Odyssey Infrared Imager (LI-COR Biosciences, Lincoln, NE). All densitometric results are reported normalized to β-actin.

### 2.4 Enzyme-linked immunosorbent assay

Blood was centrifuged at 3000 rpm for 10 min at 4°C to isolate plasma. Plasma was stored at −80°C. Plasma levels of TNF-α (R&D System catalog number RTA00, Minneapolis, MN), TGF-β (My BioSource catalog number MBS260302, San Diego, CA) and IL-10 (R&D System catalog number R1000, Minneapolis, MN) were measured using commercial ELISAs according to the manufacturer instructions.

### 2.5 Histology

Kidneys were bisected transversely and sections of thoracic aorta were dissected. Tissues were fixed in 10% formalin overnight, processed for paraffin embedding and sections onto glass slides. Tissues were stained by haematoxylin and eosin (H&E). Stained sections were visualized using an Olympus BX40 microscope (Olympus America, Melville, NY) on a bright-field setting fitted with a digital camera (Olympus DP12; Olympus America). Aortic wall thickness was measured in five different areas per aortic section using an image analysis computer program (Cellsen Standard software (Olympus) by an investigator blinded to group assignment. The average value of five measurements was reported.

### 2.6 Statistical analysis

All data are expressed as means ± SEM. For all comparisons, *p* < 0.05 was considered statistically significant. Data in control male and female SHR were compared using unpaired student-t test. BP data within each sex were analyzed using repeated measures ANOVA with Tukey’s multiple comparisons test and between group comparisons were made by 2-way ANOVA. All other data were compared using 2-way ANOVA followed by a Tukey’s multiple comparisons test. Analyses were performed using GraphPad Prism version 9.2 software (GraphPad Prism Software, La Jolla, CA).

## 3 Results

### 3.1 Adult female spontaneously hypertensive rats have greater apoptotic cell death in the kidney and aorta compared to age matched males

**FIGURE 3:**
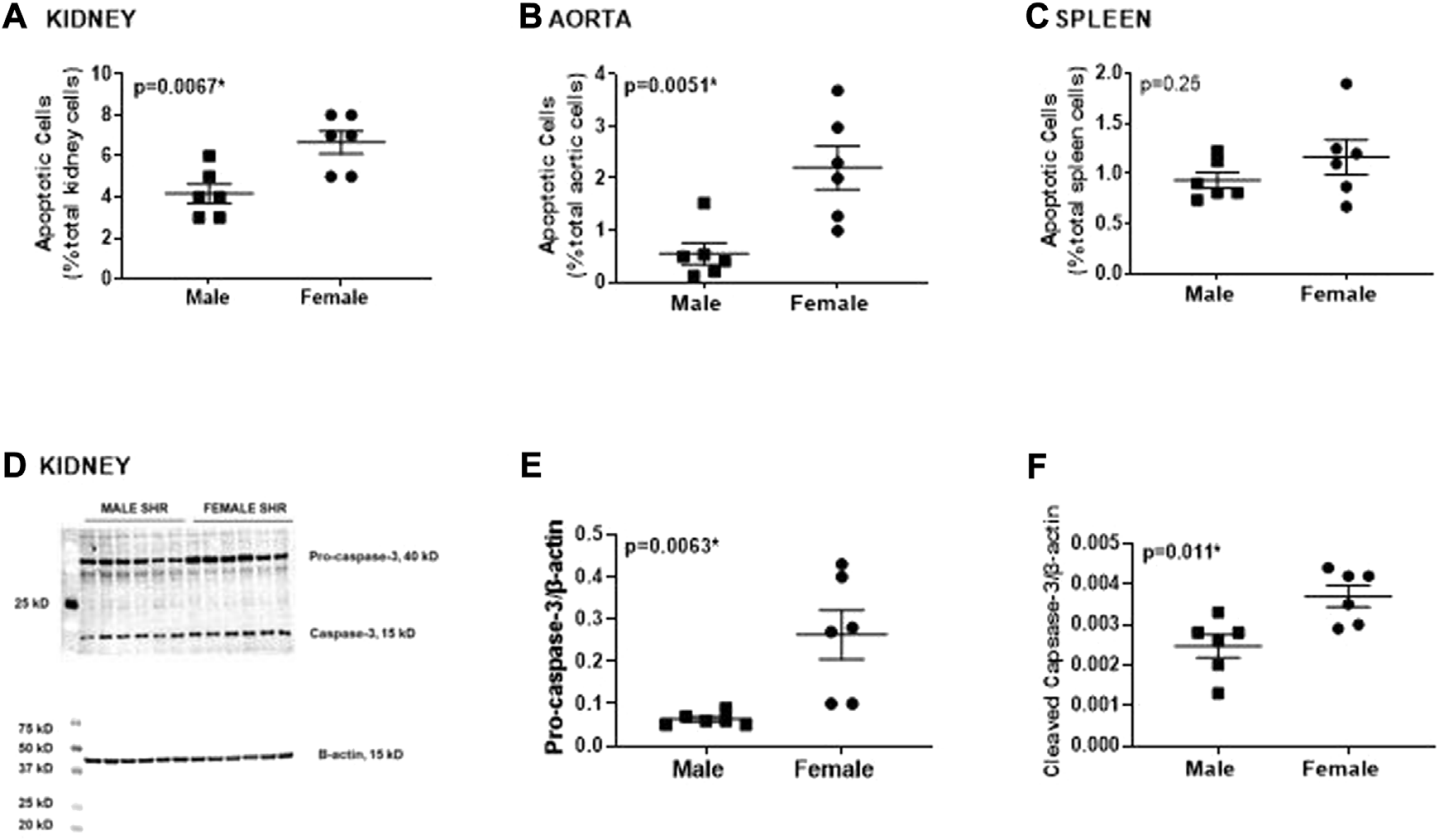
Female SHR have greater renal and aortic apoptosis compared to age matched males. Apoptotic cell death was measured by flow cytometric analysis and expressed as % of total gated cells in whole kidney [panel **(A)**], thoracic aorta [panel **(B)**] and spleen [panel **(C)**] from 13 week old male and female spontaneously hypertensive rats (SHR; *n* = 6). Protein expression of renal caspase-3 (pro and active) was measured by Western blotting. Representative blots are in panel **(D)** with mean data in panels **(E,F)**. Data were compared using unpaired student-t test.

Apoptotic cell death was measured by flow cytometric analysis of kidney, aorta and spleen isolated from young adult 13-week-old male and female SHR (Figure 3). Female SHR have significantly greater apoptosis than males in both the kidney and aorta (*p* = 0.0067 and *p* = 0.0051, respectively; Figures 3A,B). The level of apoptosis was comparable in spleen of male and female SHR (*p* = 0.25; Figure 3C).

Greater renal apoptosis in female SHR was confirmed by greater protein expression of pro- and cleaved caspase-3, a downstream key executer of apoptosis, determined by Western blotting of whole kidney homogenate (*p* = 0.0063 and *p* = 0.011, respectively; Figure 3D).

### 3.2 Inhibition of apoptosis in adult spontaneously hypertensive rats does not change blood pressure in either males or females

**FIGURE 4:**
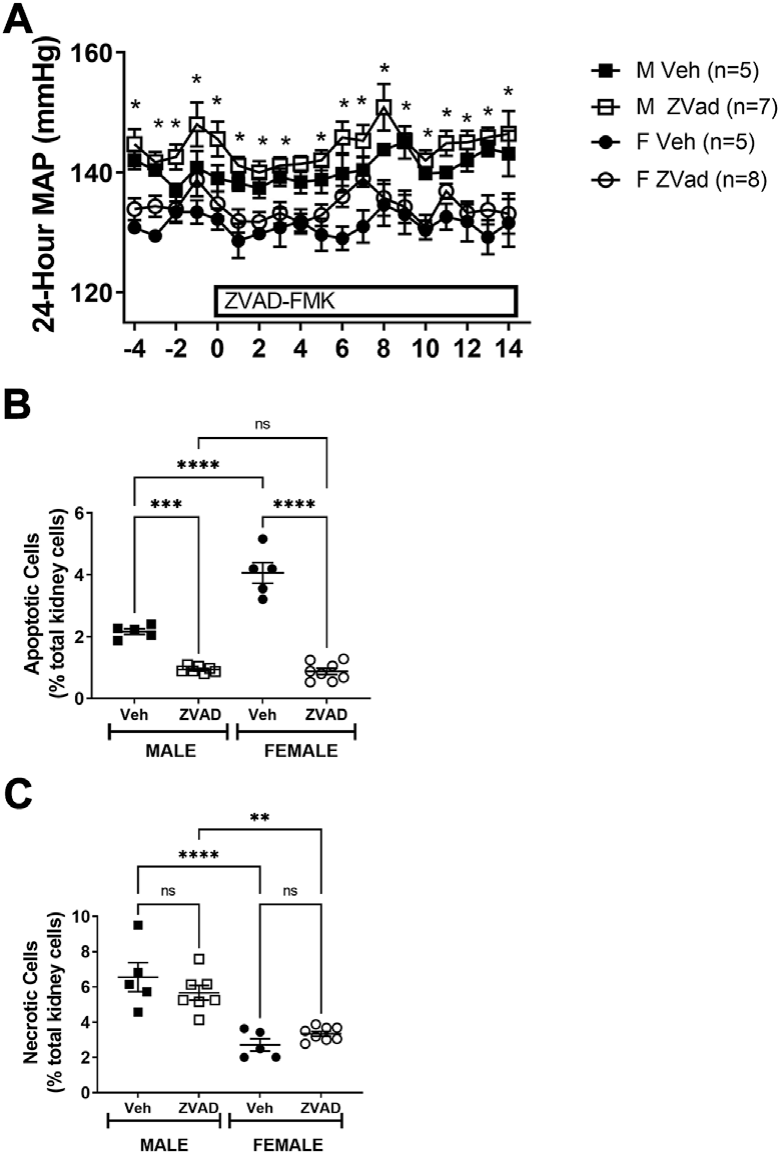
Treatment with ZVAD-FMK for 2 weeks lowers renal apoptosis, but did not change BP in male or female SHR. Mean arterial blood pressure (MAP) was measured via telemetry in male (M) and female (F) spontaneously hypertensive rats (SHR) treated with vehicle (veh) or ZVAD-FMK (ZVAD) from 13 to 15 weeks of age [panel **(A)**; *n* = 5–8]. Apoptotic [panel **(B)**] and necrotic [panel **(C)**] cell death were measured by flow cytometric analysis and expressed as % of total gated kidney cells in the same rats. MAP data within each sex were analyzed using repeated measures ANOVA with Tukey’s multiple comparisons test. Between group comparisons were made by 2-way ANOVA; * indicates *p* < 0.05 vs. female SHR. Flow cytometry data were compared using 2-way ANOVA followed by Tukey’s multiple comparisons test; **** indicates *p* < 0.0001; *** indicates *p* = 0.0001; NS indicates not significant.

To determine the contribution of apoptosis to the control of BP in established hypertension in male and female SHR, rats were treated with either vehicle or ZVAD-FMK from 13 to 15 weeks of age and BP was measured by telemetry. At baseline, BP was greater in males compared to females and BP remained higher in male SHR throughout the course of treatment (*p* < 0.05; Figure 4A). There were no differences in starting BP in males randomized to vehicle control or ZVAD (MAP in M Veh vs. M ZVAD: 140 ± 1 vs. 142 ± 2; NS; Figure 4) or in females (MAP in F Veh vs. F ZVAD: 133 ± 3 vs. 135 ± 2; NS). Treatment with ZVAD-FMK did not change BP in either male (final MAP in M Veh vs. M ZVAD: 149 ± 2 vs. 151 ± 1 mmHg, respectively; NS) or female SHR (final MAP in F Veh vs. M ZVAD: 139 ± 3 vs. 139 ± 2 mmHg, respectively; NS).

At the end of the study, kidneys were collected to confirm the effectiveness of treatment to decrease apoptosis. Treatment with ZVAD-FMK significantly lowered renal apoptosis in both male and female SHR (effect of treatment: *p* < 0.0001; Figure 4B). A more pronounced reduction of renal apoptosis in female SHR treated with ZVAD-FMK abolished the sex difference in renal apoptosis (interaction: *p* < 0.0001; effect of sex: *p* < 0.0001). The specificity of ZVAD-FMK for apoptotic cell death was further verified by measurement of renal necrotic cell death at the end of the treatment period. Renal necrosis was not affected by treatment with ZVAD-FMK in male or female SHR (effect of treatment: *p* = 0.74; interaction: *p* = 0.22; Figure 4C) and consistent with our previous publication (Abdelbary et al., 1979), male SHR had greater renal necrosis compared to females (effect of sex: *p* < 0.0001; Figure 4C).

### 3.3 Inhibition of apoptosis for 2 weeks in adult spontaneously hypertensive rats with established hypertension decreased Th17 cells in the male, but not female kidney

**FIGURE 5:**
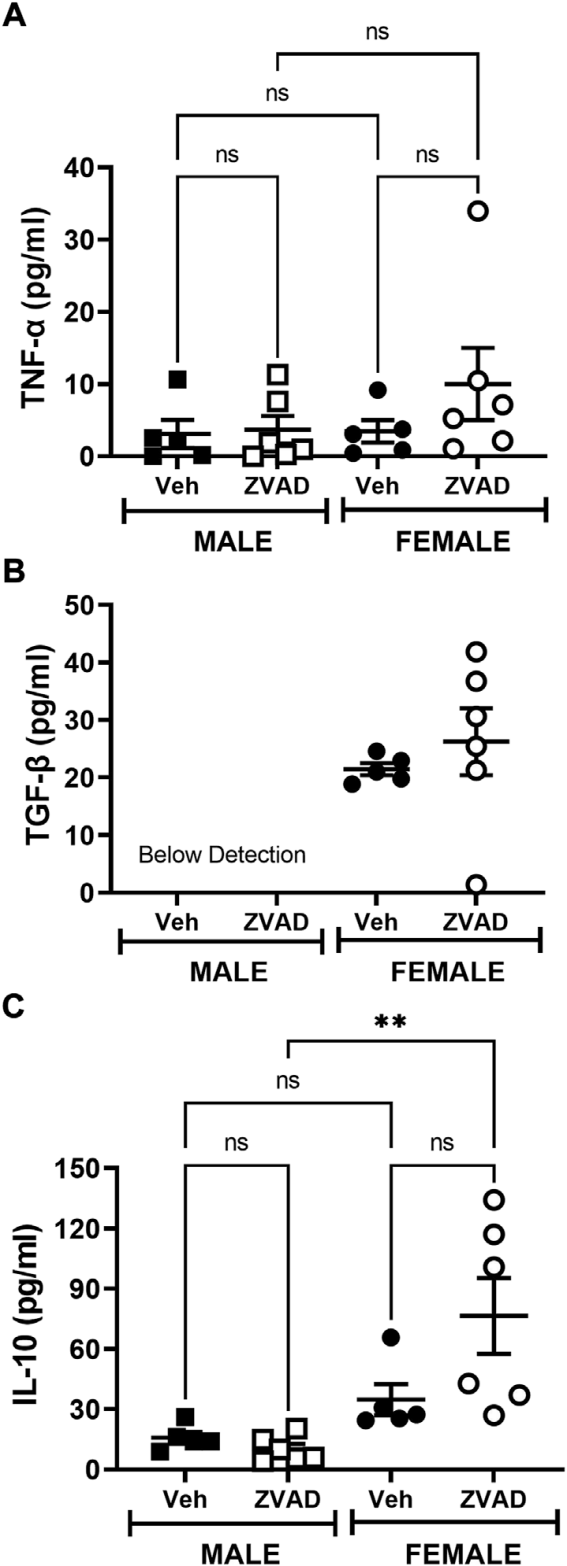
Treatment with ZVAD-FMK for 2 weeks does not increase circulating pro-inflammatory cytokines in male or female SHR. Circulating levels of TNF-α [panel **(A)**], TGF-β [panel **(B)**], and IL-10 [panel **(C)**] were measured by ELISA in the plasma isolated from male and female spontaneously hypertensive rats (SHR) treated with vehicle (veh) or ZVAD-FMK (ZVAD) from 13 to 15 weeks of age (*n* = 5–6). Data were compared using 2-way ANOVA followed by Tukey’s multiple comparisons test; ** indicates *p* < 0.01; NS indicates not significant.

**FIGURE 6:**
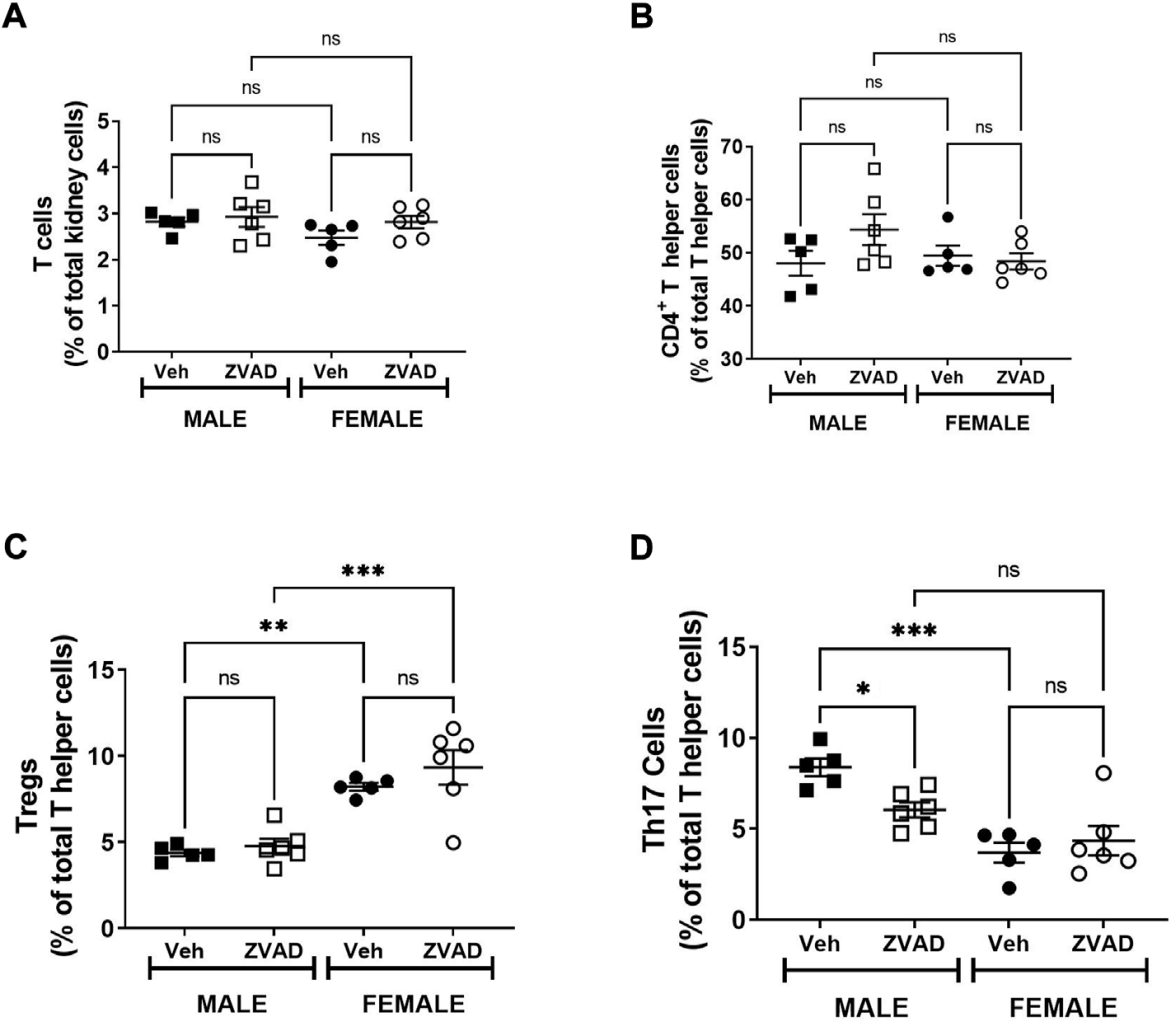
Treatment with ZVAD-FMK for 2 weeks decreases Th17 cells in male SHR. T cells were measured in kidneys isolated from male and female spontaneously hypertensive rats (SHR) treated with vehicle (veh) or ZVAD-FMK (ZVAD) from 13 to 15 weeks of age (*n* = 5–6). The following T cells were measured: total CD3^+^ T cells [panel **(A)**], CD4^+^ T helper cells [panel **(B)**], T regulatory cells [Tregs; CD3^+^CD4^+^FOXP3^+^; panel **(C)**] and T helper [Th17 cells (CD3^+^CD4^+^ROR- γ^+^; panel **(D)**]. CD3^+^ T cells were expressed as % total renal cells. CD4^+^ T cells were expressed as % total CD3^+^ T cells. Tregs and Th17 cells were expressed as % CD3^+^CD4^+^ T cells. Data were compared using 2-Way ANOVA followed by Tukey’s multiple comparisons test; *** indicates *p* < 0.001; ** indicates *p* < 0.01; NS indicates not significant.

Apoptotic cell death is an anti-inflammatory form of cell death (Dellepiane et al., 2020). To assess the impact of apoptosis on the immune profile, we assessed circulating levels of the pro-inflammatory cytokines TNF-α and TGF-β and the anti-inflammatory cytokine IL-10, as well as the renal T cell profile. The kidney was chosen because there are sex differences in both renal apoptosis and the renal T cell profile (Abdelbary et al., 1979; Tipton et al., 2012). Two weeks of ZVAD-FMK treatment did not change circulating levels of TNF-α (Interaction: *p* = 0.36; effect of sex: *p* = 0.30; effect of treatment: *p* = 0.28, Figure 5A) or TGF-β (Interaction: *p* = 0.55; effect of sex: *p* < 0.0001; effect of treatment: *p* = 0.40, Figure 5B). Interestingly, circulating IL-10 levels increased in female, but not male SHR (Interaction: *p* = 0.048; effect of sex: *p* = 0.0013; effect of treatment: *p* = 0.12, Figure 5C). Treatment with ZVAD-FMK for 2 weeks did not alter renal CD3^+^ T cells (Interaction: *p* = 0.49; effect of sex: *p* = 0.18; effect of treatment: *p* = 0.19, Figure 6A), CD4^+^ T cells (Interaction: *p* = 0.12; effect of sex: *p* = 0.33; effect of treatment: *p* = 0.26, Figure 6B) or Tregs (Interaction: *p* = 0.56; effect of sex: *p* < 0.0001; effect of treatment: *p* = 0.23, Figure 6C) in male or female SHR. However, ZVAD decreased the percentage of pro-inflammatory Th17 cells in kidneys of male, but not female SHR abolishing the sex difference (Interaction: *p* = 0.023; effect of sex: *p* < 0.0001; effect of treatment: *p* = 0.18; Figure 6D). Consistent with previous reports, female SHR had higher circulating levels of TGF-β (levels were below detection in males) and IL-10 and a greater percentage of renal Tregs compared to males (Tipton et al., 2017; Gillis et al., 2020).

### 3.4 Inhibition of apoptosis does not affect the development of hypertension in male or female spontaneously hypertensive rats

Two weeks of inhibition of apoptosis after hypertension was established did not alter BP. However, 2 weeks may have been insufficient to impact BP or the renal T cell profile. Therefore, additional studies randomized 6 week old pre-hypertensive male and female SHR to vehicle or receive ZVAD-FMK treatment for 6 weeks and systolic BP was monitored weekly by tail-cuff. Based on sex differences in renal apoptosis in adulthood, additional studies measured renal apoptosis in 6 week old male and female SHR, prior to the development of hypertension. Even before the development of hypertension, females have greater renal apoptosis than males (% total renal cells: 1.9 ± 0.2 vs. 0.7 ± 0.1, *p* < 0.0005).

**FIGURE 7:**
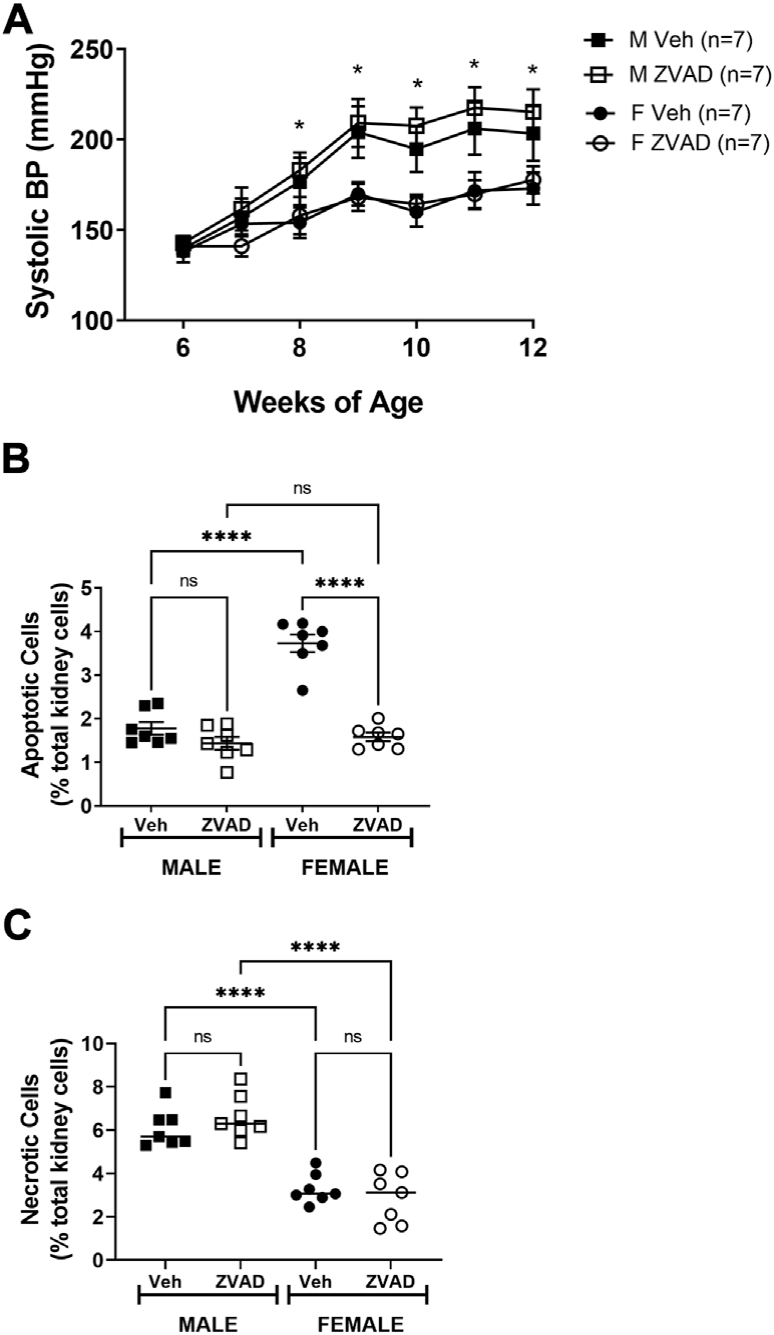
Treatment with ZVAD-FMK for 6 weeks starting prior to the development of hypertension did not change BP in male or female SHR. Systolic BP was measured by tail-cuff in male (M) and female (F) spontaneously hypertensive rats (SHR) treated with vehicle (veh) or ZVAD-FMK (ZVAD) from 6 to 12 weeks of age [*n* = 7; panel **(A)**]. Apoptotic [panel **(B)**] and necrotic [panel **(C)**] cell death were measured by flow cytometric analysis and expressed as % of total gated kidney cells in the same rats. BP data within each sex were analyzed using repeated measures ANOVA with Tukey’s multiple comparisons test and between group comparisons were made by 2-way ANOVA; * indicates *p* < 0.05 vs. female SHR. Flow cytometry data were compared using 2-way ANOVA followed by Tukey’s multiple comparisons test; **** indicates *p* < 0.0001; NS indicates not significant.

At baseline, BP was comparable between male and female SHR and between rats randomized to vehicle or ZVAD treatment (systolic BP in mmHg: M Veh 140 ± 4; M ZVAD 143 ± 4; F Veh 138 ± 6; F ZVAD 141 ± 4; NS; Figure 7A). BP increased progressively with age in both male and female SHR (*p* < 0.05) and males had a greater BP than females beginning at 8 weeks of age (*p* < 0.05). Treatment with ZVAD-FMK did not change BP in male (final systolic BP in M Veh vs. M ZVAD: 203 ± 15 vs. 215 ± 12 mmHg; respectively; NS) or female SHR (final systolic BP in F Veh vs. F ZVAD: 173 ± 8 vs. 178 ± 8 mmHg; respectively; NS).

Following 6 weeks of treatment, kidneys were collected to assess the effectiveness of ZVAD-FMK to lower apoptosis. ZVAD-FMK significantly decreased renal apoptosis in male and female SHR (effect of treatment: *p* < 0.0001; Figure 7B) and the reduction of renal apoptosis was more pronounced in females (interaction: *p* < 0.0001; effect of sex: *p* < 0.0001). Renal necrosis was also measured following 6 weeks of treatment with ZVAD-FMK to assess the selectivity of ZVAD-FMK for apoptosis. Treatment of ZVAD-FMK did not change renal necrosis in male or female SHR (effect of treatment: *p* = 0.71; Figure 7C).

### 3.5 Inhibition of apoptosis for 6 weeks did not alter the renal T cell profile in either sex

**FIGURE 8:**
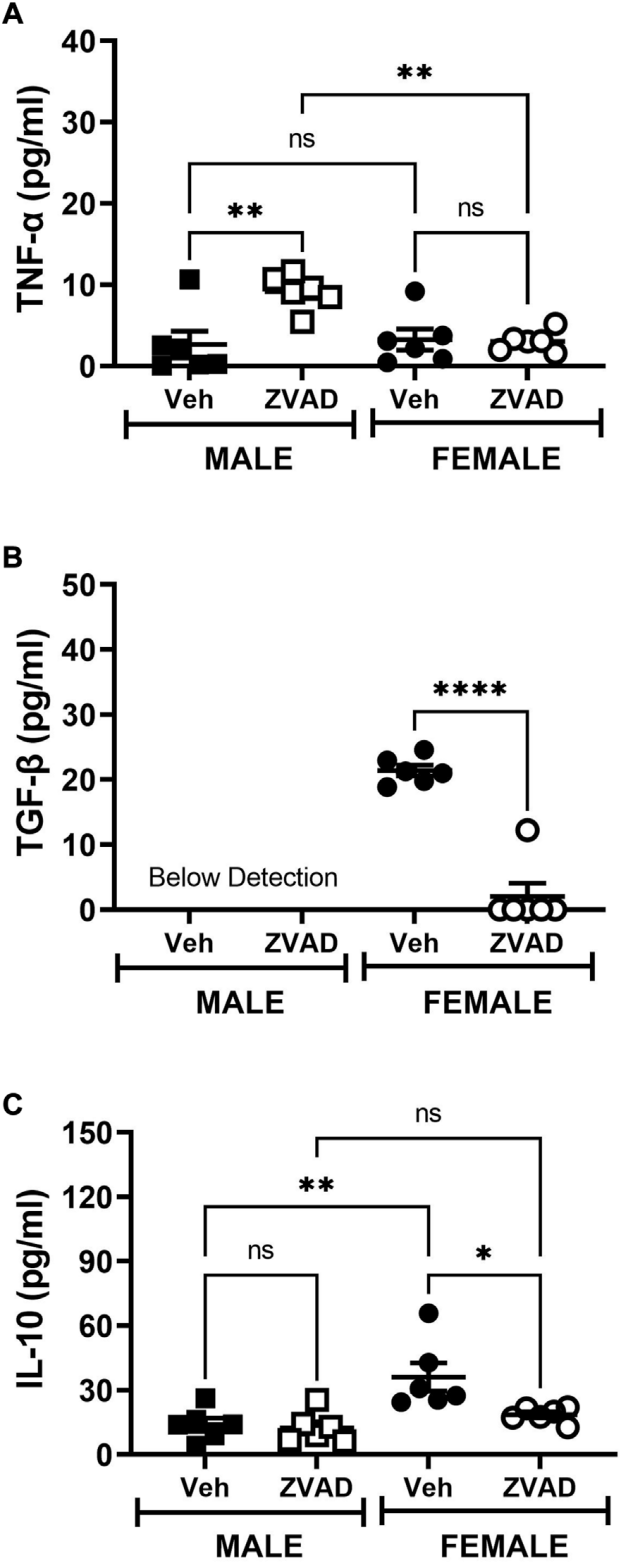
Treatment with ZVAD-FMK for 6 weeks does not increase circulating pro-inflammatory cytokines in male or female SHR. Circulating levels of TNF-α [panel **(A)**], TGF-β [panel **(B)**], and IL-10 [panel **(C)**] were measured by ELISA in the plasma isolated from male and female spontaneously hypertensive rats (SHR) treated with vehicle (veh) or ZVAD-FMK (ZVAD) from 6 to 12 weeks of age (*n* = 6). Data were compared using 2-way ANOVA followed by Tukey’s multiple comparisons test; **** indicates *p* < 0.0001; ** indicates *p* < 0.01; * indicated *p* < 0.05; NS indicates not significant.

**FIGURE 9:**
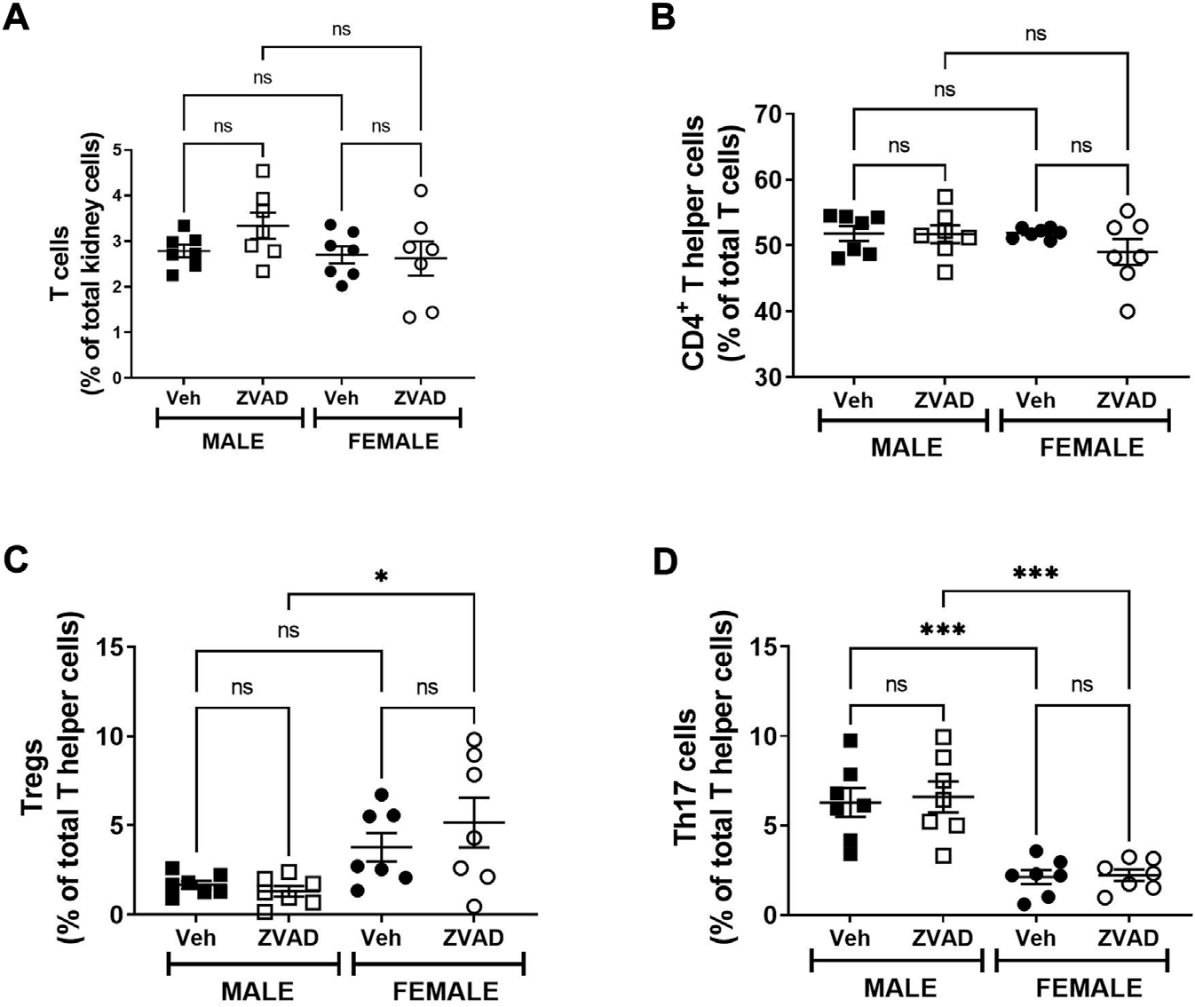
Treatment with ZVAD-FMK for 6 weeks did not change either splenic or renal T cell profile. T cells were measured in kidneys isolated from male and female spontaneously hypertensive rats (SHR) treated with vehicle (veh) or ZVAD-FMK (ZVAD) from 6 to 12 weeks of age (*n* = 7). The following T cells were measured: total CD3^+^ T cells [panel **(A)**], CD4^+^ T helper cells [panel **(B)**], T regulatory cells [Tregs; CD3^+^CD4^+^FOXP3^+^; panel **(C)**] and T helper (Th17 cells [CD3^+^CD4^+^ROR- γ^+^; panel **(D)**]. CD3^+^ T cells were expressed as % total renal cells. CD4^+^ T cells were expressed as % total CD3^+^ T cells. Tregs and Th17 cells were expressed as % CD3^+^CD4^+^ T cells. Data were compared using 2-Way ANOVA followed by Tukey’s multiple comparisons test; *** indicates *p* < 0.001; * indicated *p* < 0.05; NS indicates not significant.

TNF-α, TGF-β, and IL-10 were measured in plasma following 6 weeks of ZVAD treatment. TNF-α levels increased in male SHR (Interaction: *p* = 0.009; effect of sex: *p* = 0.029; effect of treatment: *p* = 0.014, Figure 8A), while TGF-β (Interaction: *p* = 0.0001; effect of sex: *p* = 0.0001; effect of treatment: *p* = 0.0001, Figure 8B) and IL-10 (Interaction: *p* = 0.05; effect of sex: *p* = 0.0018; effect of treatment: *p* = 0.025, Figure 8C) decreased only in females. T cells were also assessed in the kidney following 6 weeks of treatment with ZVAD-FMK. Similar to the 2-week treatment, ZVAD-FMK did not change the percentage of CD3^+^ T cells (effect of treatment: *p* = 0.31, effect of sex: *p* = 0.18, effect of treatment: *p* = 0.38; Figure 9A), CD4^+^ (effect of treatment: *p* = 0.35, effect of sex: *p* = 0.33, effect of treatment: *p* = 0.31; Figure 9B), or Tregs (effect of treatment: *p* = 0.2, effect of treatment: *p* = 0.4; Figure 9C). In contrast to 2 weeks of ZVAD treatment, Th17 cells were not impacted by 6 weeks of ZVAD treatment (effect of treatment: *p* = 0.9, effect of treatment: *p* = 0.8; Figure 9D).

**FIGURE 10:**
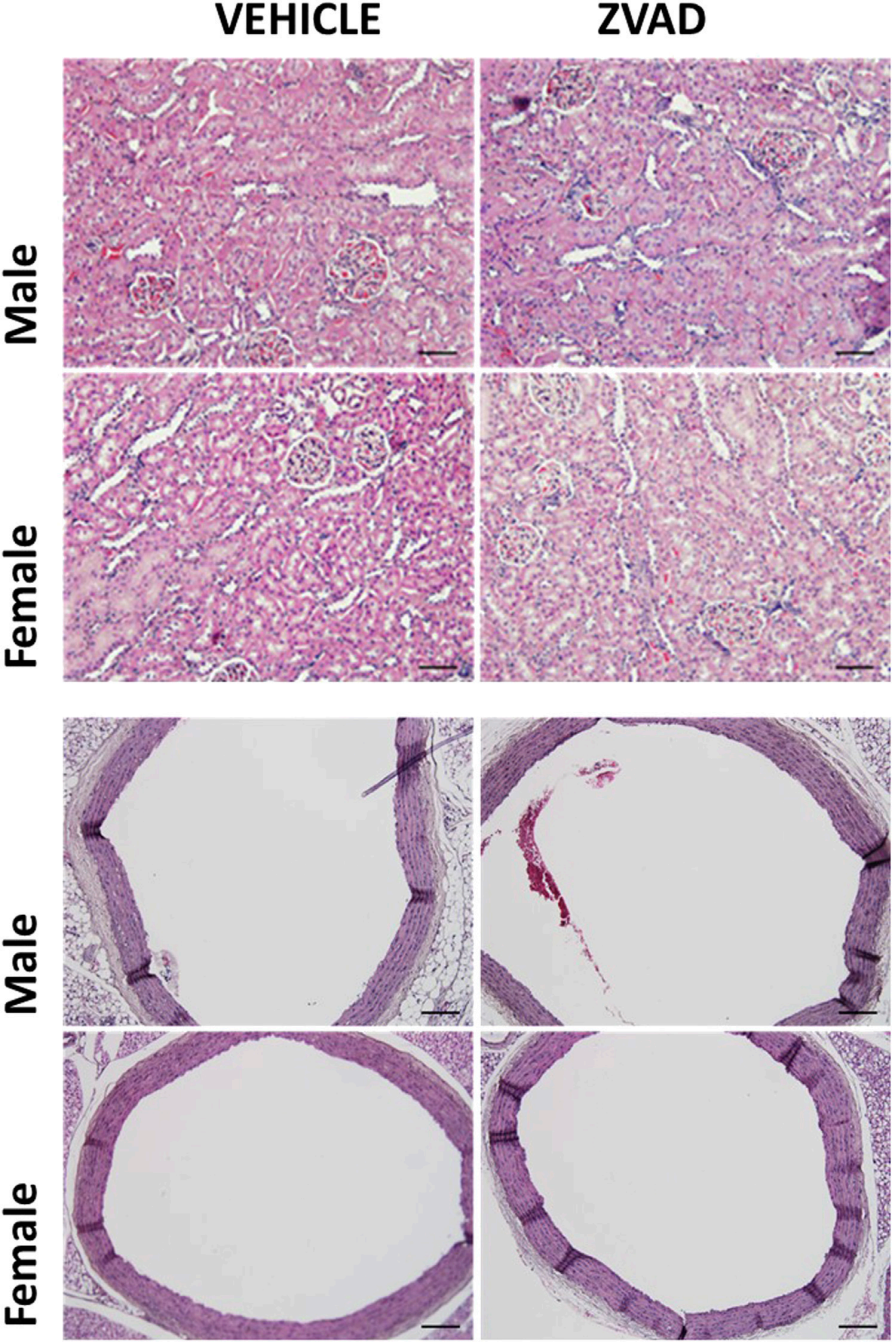
Treatment with ZVAD-FMK for 6 weeks did not change either aortic or renal structure. Aorta and kidney were harvested and formalin fixed from male and female spontaneously hypertensive rats (SHR) treated with vehicle (veh) or ZVAD-FMK (ZVAD) from 6 to 12 weeks of age (*n* = 3).

Since there were noted sex differences in apoptosis in the aorta of male and female SHR, additional studies also determined if 6 weeks of ZVAD-FMK altered aortic structure (Figures 10A,B). There were no differences in aortic histology and wall thickness was unaltered by ZVAD treatment in either male (control vs. ZVAD: 208 ± 6 vs. 206 ± 5 µm) or female SHR (control vs. ZVAD: 208 ± 4 vs. 204 ± 10 µm). Renal histology was also assessed, and consistent with the findings in the aorta, there were not histological changes with VZAD treatment (Figure 10C).

## 4 Discussion

While numerous studies have reported increases in apoptotic cell death in multiple organs during hypertension (Fortuño et al., 1979; Hamet et al., 1979; Jiang et al., 2013), the main finding of the current study is that apoptosis does not contribute to BP control in SHR. In addition, despite a greater level apoptotic cell death in female SHR when compared to males, apoptotic cell death does not contribute to sex differences in either BP or the renal T cell profile.

Apoptosis has been detected in multiple tissues in SHR including the heart, kidney, and blood vessels (Hamet et al., 1996). High levels of ventricular apoptosis is a characteristic feature in animal models of essential hypertension, including SHR (Díez et al., 1979; Hamet et al., 1979; Li et al., 1997), and increased vascular apoptosis has been reported in male DOCA salt hypertension (Sharifi and Schiffrin, 1997), SHR (Sharifi and Schiffrin, 1998), and Ang II induced hypertensive rats (Diep et al., 1979). Apoptosis is also higher in hearts of patients with essential hypertension compared to normotensive patients at biopsy (González et al., 1979). Apoptosis of vascular smooth muscle cells contributes to vascular remodeling (Bennett, 1999), and apoptosis has been suggested as a therapeutic target to prevent hypertension-associated changes in the vasculature (deBlois et al., 1979; Intengan and Schiffrin, 1979; Stead et al., 2000). Apoptosis has been speculated to initially serve as a regulatory mechanism that contributes to enhanced repair and maintenance of homeostasis in the kidney following renal injury in response to various pathological insults (Cao et al., 2000; Bonnet et al., 2001). However, excessive and persistent apoptosis leads to renal fibrosis and disruption normal kidney function (Thomas et al., 1998; Yeboah et al., 2016). While the specific cell types most susceptible to cell death in the hypertensive kidney of either sex remain to be fully identified and characterized, tubular epithelial cells from SHR have been reported to be more susceptible to apoptosis compared to those from normotensive WKY (Soto et al., 2004). In addition, the development of hypertension is associated with an increase in apoptosis in renal glomerular and tubular epithelial cells in a rat model of intrauterine growth restriction (Vehaskari et al., 2001).

`However, the relationship between apoptosis and hypertension has remained uncertain. It has been questioned whether apoptosis precedes the development of hypertension or is a consequence of high BP. An investigation of cardiomyocyte apoptosis in male SHR reported increases with age, with maximal apoptosis at sexual maturation (8 weeks of age). Interestingly, apoptosis remained elevated vs. normotensive controls until 16 weeks of age then decreased, despite consistently elevated BP. Suggesting that cardiomyocyte apoptosis in SHR is independent of high BP, a conclusion supported by numerous additional studies (Fortuño et al., 1979; Hamet et al., 1979; Moreau et al., 1979; Li et al., 1997; Liu et al., 2000).

The majority of studies in the literature that have assessed apoptosis in hypertension have focused on males. However, consistent with previous reports, apoptotic cell death was greater in the aorta and kidney of female SHR compared to males. Importantly, sex differences in renal apoptosis were apparent even in 6 week old male and female SHR, prior to the development of sexual maturation or hypertension. Several studies report that female cells are programmed to attain better homeostasis and respond to stress in a more benign and less debilitating manner when compared to cells from males, which tend to be more prone to stress-mediated cell death (Du et al., 2004; Ortona et al., 2014). The finding of sex differences in apoptosis in young SHR further supports this notion. Previous studies have shown that neurons and bone marrow-derived macrophages isolated from female mice tend to die predominantly via apoptosis when subjected to stress, while cells from males are more likely to undergo necrosis (Du et al., 2004; Jog and Caricchio, 2013). Moreover, greater apoptosis in females is associated with less damage and better prognosis in animal models of ischemic stroke compared to males (Li et al., 2005; Lang and McCullough, 2008), suggesting that apoptotic cell death may contribute to the cardiovascular protection typically afforded young females vs. males.

Despite greater apoptosis in females, and well-established sex differences in hypertension in SHR where females have a lower BP compared to age-matched male SHR, inhibition of apoptosis did not alter BP in either males or females. Consistent with our results, treatment of male SHR with a combination of ZVAD-FMK and the angiotensin receptor blocker losartan did not change in BP despite an improvement in vascular remodeling by ZVAD-FMK (Marchand et al., 2003). As we did not measure vascular function or remodeling in the current study, we cannot rule out that inhibition of apoptosis affected cardiovascular or renal function outside of a change in BP. Interestingly, ZVAD-FMK also attenuates the prolonged BP-lowering effect of losartan in male SHR. BP remained lowered in male SHR treated with ZVAD-FMK despite discontinuation of losartan treatment. Therefore, while there was not a direct BP lowering effect of ZVAD-FMK, inhibition of apoptosis extended the BP-lowering effect of anti-hypertensive treatment (Baumann et al., 2009). The current study was the first to treat hypertensive females with ZVAD-FMK. Indeed, since females have greater apoptosis, we anticipated that ZVAD-FMK may have a more pronounced effect in females. While this was not what we found, treatment did decrease apoptosis. Future studies will challenge rats to determine if inhibition of apoptosis influences the BP response to agents that either increase or decrease BP. Alternatively, BP regulation is highly complex process involving numerous organ systems, therefore a longer treatment time may be needed before a change in BP takes place. Apoptosis is a physiological process that is well-tolerated by the body, therefore inhibition of apoptosis might take a long time before the development of physiological changes.

Inhibition of apoptosis by ZVAD-FMK has been reported to increase necrotic cell death (Wu et al., 2011; Dong et al., 2016; Koike et al., 2019) and pretreatment of renal tubular cells with ZVAD-FMK significantly increased Ang II-induced necrotic cell death *in vitro* (Zhu et al., 2020). Since we previously reported that greater necrosis in male SHR contributes to the development of hypertension (Abdelbary et al., 1979), we measured necrosis in ZVAD-FMK treated rats and found no changes with treatment. However, we did not measure all other forms of cell death and therefore cannot conclude that additional forms of cell death may be increased and balance the effect of decreasing apoptosis to obscure any potential BP or inflammatory effects. In particular, assessment of pyroptosis would be of interest. There is increasing evidence implicating a role for pyroptosis in hypertension, but few studies have directly examined this relationship (Ulrich et al., 2020; De Miguel et al., 2021).

How a cell dies impacts the development of an immune response. In contrast to other forms of cell death, apoptosis does not induce a robust inflammatory response and in many cases, clearance of apoptotic cells by phagocytic cells such as macropahges (Szondy et al., 2017) results in the release of anti-inflammatory molecules such as TGFβ and IL-10 to enhance tissue repair and maintain homeostasis (Fadok et al., 1998; Szondy et al., 2017). Macrophages are central regulators in T cell activation. Indeed, whether macrophages activate or inhibit T cells is dependent on macrophage phenotype, costimulatory molecules, and the cytokine milieu (Guerriero, 2019). Based on our interest in BP control and the known role for T cells in contributing to the development of hypertension, we measured circulating cytokines and renal T cells to determine if sex differences in apoptosis contributed to sex differences in inflammation. The hypothesis was that greater apoptosis in female SHR would contribute to the more anti-inflammatory profiles vs. males such that inhibition of apoptosis would increase pro-inflammatory cytokines and T cells, particularly in females. In contrast, 2 weeks of ZVAD treatment increased circulating levels of the anti-inflammatory cytokine IL-10 only in females and decreased Th17 cells only in males, supporting a pro-inflammatory role for apoptosis. However, this was not supported by longer treatment with ZVAD-FMK. Apoptosis is of fundamental importance for the development and selection of lymphocytes in the thymus (Zhang et al., 2005). Following exposure to its cognate antigens, T cells undergo activation and proliferation (expansion) followed by contraction through apoptotic cell death of approximately 90% of activated T cells leaving behind long-lived, memory T cells (Adams et al., 2020). The current study did not assess T cell activation, memory T cells, or macrophage phenotype or activity. Moreover, several studies have shown that it takes weeks to months to establish a population of tissue-resident T cells (Harty and Badovinac, 2008). While apoptosis is important for the formation of tissue-resident T cells, it may take longer than 6 weeks following inhibition of apoptosis for a change in the frequency of T cells to be observed.

## Data Availability

The original contributions presented in the study are included in the article/Supplementary Material; further inquiries can be directed to the corresponding author.
